# Cardiac Macrophages Exhibit Dynamic Heterogeneity and Functional Specialization During Experimental Autoimmune Myocarditis

**DOI:** 10.3390/cells15121110

**Published:** 2026-06-19

**Authors:** Monika Stefanska, Marta Kot, Damian Koterba, Joanna Zeyland

**Affiliations:** 1 Department of Biochemistry and Biotechnology, Poznan University of Life Sciences, 60-637 Poznan, Poland; joanna.zeyland@puls.edu.pl; 2 Department of Transplantation, Faculty of Medicine, Institute of Paediatrics, Jagiellonian University Medical College, 30-663 Krakow, Poland; marta.kot@uj.edu.pl; 3 Department of Computer Science, Faculty of Computer Science and Mathematics, Cracow University of Technology, 31-155 Krakow, Poland; damian.koterba@student.pk.edu.pl

**Keywords:** experimental autoimmune myocarditis, cardiac macrophages, cardiac remodelling, inflammation, snRNA-sequencing, cell–cell communication analysis

## Abstract

Autoimmune myocarditis frequently progresses to inflammatory cardiomyopathy through dysregulated immune–stromal interactions. This study employs single-nuclei RNA-sequencing (snRNA-seq) to profile 46,233 cardiac nuclei from the experimental autoimmune myocarditis (EAM) mouse model at four timepoints: day 0 (healthy), day 14 (inflammation), day 21 (acute inflammation), and day 40 (late cardiac remodelling). Single-nuclei RNA profiling identified 18 transcriptionally distinct cell populations. Global cell–cell communication analysis revealed a dramatic peak of intercellular signalling at day 14 (5907 interactions), with fibroblast subpopulations and macrophages as dominant hubs, followed by partial resolution at day 21 (2264 interactions) and renewed remodelling at day 40 (4862 interactions). Subclustering of the macrophage compartment identified five subpopulations: Mac-TLF, Mac-MHCII, Mac-rMHCII, Mac-ResL, and Classical Monocytes. Tissue-resident macrophages (Mac-TLF, CCR2-) dominated at healthy state (~55%) but were rapidly depleted at day 14, coinciding with a dramatic influx of recruited CCR2^+^ macrophages (Mac-rMHCII), which expanded to over 70% of the compartment and maintained dominance through day 40. At inflammation (day 14), the expanded Mac-rMHCII subpopulation displayed a strongly pro-inflammatory signature (*Il1b*, *Stat2*, *Parp14*, *Apoe*), and the overall macrophage compartment was enriched for cytokine response, Fc-gamma receptor, and Notch signalling pathways, while downregulating homeostatic and mitochondrial metabolic programmes, potentially contributing to impaired efferocytosis and cardiomyocyte dysfunction. Macrophage-centred communication networks expanded markedly at day 14 (1047 interactions), with resting fibroblasts (FB-R) as the primary signalling partner, driving pro-inflammatory stromal activation marked by upregulation of *Ccl2*, *Ccl7*, and *Csf2*. Intra-macrophage subcluster communication also intensified at this timepoint (447 interactions). These findings delineate the temporal and functional heterogeneity of cardiac macrophages during EAM progression and identify key immune–stromal interactions driving pathological cardiac remodelling. The coexistence of pro-inflammatory and transitional reparative macrophage subsets highlights the limitations of broad immunosuppression and supports precision strategies targeting CCR2-mediated recruitment, the SPP1 signalling axis, and macrophage–fibroblast crosstalk as therapeutic avenues in myocarditis and its progression.

## 1. Introduction

Myocarditis is an inflammatory disease of the heart that can lead to dilated cardiomyopathy (DCM) or heart failure [1,2]. It is defined as an acute inflammation of the myocardial tissue caused by direct pathogen-induced damage and/or an excessive immune response [3]. The etiology of the disease is diverse and includes both infectious agents (e.g., viruses such as *Coxsackievirus*) and non-infectious factors (e.g., autoimmune processes) [2,3,4]. The progression of myocarditis encompasses a broad spectrum of clinical changes, from the acute inflammatory phase, through healing processes, to a potential transition into chronic inflammatory cardiomyopathy [3]. This cardiomyopathy is characterized by progressive tissue fibrosis, adverse remodelling, and a decline in contractile function [2,5]. The progression of myocarditis to a dilated cardiomyopathy is markedly influenced by TGF-β signalling [6].

Macrophages, which constitute a highly heterogeneous cell population, are key mediators in both maintaining homeostasis and the pathogenesis of myocardial injury [7,8]. In the heart, two main lineages of macrophages with distinct origins and functions are distinguished: tissue-resident macrophages (TRMs; typically CCR2-), predominantly derived from embryonic development, and recruited monocyte-derived macrophages (CCR2^+^), which infiltrate from the bloodstream in response to injury [7,8,9,10]. In a healthy heart, tissue-resident macrophages (TRMs) (including subsets expressing *Timd4*, *Lyve1*, and *Folr2*, collectively termed TLF^+^) constitute the vast majority (approximately 70%) of the cardiac immune compartment and are responsible for maintaining tissue homeostasis [9]. However, acute inflammation drastically disrupts this balance. In response to acute injury, this primary resident population undergoes rapid depletion, declining by roughly 70% in the injury zone within the first few days. Their localized loss is immediately followed by a massive influx of pro-inflammatory, monocyte-derived macrophages (CCR2^+^) recruited from the circulation [8,9,10].

During the course of experimental autoimmune myocarditis (EAM), a significant increase in the number of pro-inflammatory *Ly6Chi* monocytes and recruited CCR2^+^ macrophages is observed throughout the cardiac tissue [7,8,10]. Beyond immune regulation, cardiac macrophages directly interact with cardiomyocytes. Under physiological conditions, this crosstalk is vital for maintaining electrical stability and metabolic homeostasis through the efferocytosis of damaged mitochondria [11,12]. However, during EAM, dysregulated macrophage–cardiomyocyte interactions impair contractility and exacerbate tissue damage [3,8,10].

The functional polarization of cardiac macrophages is closely linked to their physical localization within specialized tissue niches, where they integrate diverse cues from adjacent stromal cells [8]. Within these niches, macrophage-dependent signalling pathways drive adverse remodelling through intense crosstalk with cardiac fibroblasts [13]. During the acute inflammatory phase of EAM, elevated levels of interleukin-17A stimulate extensive cardiac fibrosis and irregular collagen deposition [8,10,13]. This signalling, amplified by macrophage-derived cytokines such as IL-1β and TNF-α, transforms cardiac fibroblasts into active immune-like inflammatory hubs secreting CSF2 and CCL2 [13,14]. Recent spatial transcriptomics studies emphasize that fibroblast activation and their transition into myofibroblasts strictly depend on their physical colocalization with infiltrating immune cells, creating a self-amplifying inflammatory niche [14]. Specifically, SPP1-expressing (SPP1^+^) macrophages play a crucial role in amplifying this damage by inducing fibroblasts to adopt a highly pro-inflammatory CCL2^+^ CCL7^+^ phenotype [15].

Currently, standard non-specific immunosuppressive therapies risk silencing reparative macrophage populations, highlighting the critical need to map specific functional subsets and their spatial niches for precision immunomodulation [3,10,16]. To further elucidate these complex immune–stromal interactions, this study investigates the temporal dynamics and functional specialization of cardiac macrophages during the progression of experimental autoimmune myocarditis to late cardiac remodelling. By utilizing single-nuclei RNA-sequencing (snRNA-sequencing) across key disease stages, we aim to delineate the subset-specific roles of macrophages in inflammation and tissue remodelling. Ultimately, our research seeks to map these critical cellular pathways and highlight potential avenues for precision immunomodulation.

## 2. Materials and Methods

### 2.1. Ethics Statement

All animal procedures complied with Polish law and were approved by the Local Ethics Committee for Animal Experiments in Poznań (approval no. 63/2024), in accordance with Directive 2010/63/EU of the European Parliament and of the Council on the protection of animals used for scientific purposes.

### 2.2. Animals and Housing

Wild-type BALB/c mice (6–8 weeks old) were housed in individually ventilated cages at 22 ± 2 °C, 55 ± 10% humidity, 15 air changes/h, and a 12 h light/dark cycle (no daylight exposure), with ad libitum food and water.

### 2.3. Experimental Autoimmune Myocarditis (EAM) Induction

EAM was induced in male BALB/c mice (6–8 weeks of age) by subcutaneous injection of 200 µg α-MyHC614-634 peptide (Ac-RSLKLMATLFSTYASADR-OH; Caslo, Lyngby, Denmark) emulsified 1:1 with Complete Freund’s Adjuvant on days 0 and 7. Each experimental group consisted of eight males. At endpoint, mice were anesthetized with ketamine/xylazine (90/10 mg/kg i.p.) and isoflurane, then euthanized by cardiac puncture for blood/tissue collection and weighing.

For single-nucleus RNA-sequencing (snRNA-seq), nuclei were isolated from cardiac tissue of four mice per group. Individual samples were processed using on-chip multiplexing to allow sample demultiplexing post-sequencing while minimizing batch effects. The target nuclei recovery per sample was 5000 nuclei.

### 2.4. Nuclei Isolation and FACS

Nuclei were isolated as previously described [17]. Snap-frozen hearts were homogenized in HB buffer, filtered (40 µm strainer), and centrifuged (500× *g*, 5 min, 4 °C). Nuclear pellets were resuspended in storage buffer for FACS (Sony MA900, Sony Biotechnology Inc., San Jose, CA, USA, 100 µm nozzle) after staining with 7-AAD (0.25 µg; Thermo Fisher Scientific, Waltham, MA, USA). Gating excluded debris/doublets, yielding >99% pure singlets (2 × 10^5^ nuclei/sample).

### 2.5. Library Preparation and Sequencing

Nuclei (approximately 1000/µL) were loaded on Chromium X for GEMs using GEM-X Universal 3′ Gene Expression v4 4-plex (10× Genomics, Pleasanton, CA, USA, PN-1000779). Libraries were sequenced on Illumina NovaSeq X (Illumina, Inc., San Diego, CA, USA, paired-end, dual indexing, 28-8-0-91 cycles; 50,000 reads/nuclei).

### 2.6. Single-Nucleus RNA-Sequencing Analysis

#### 2.6.1. Pre-Processing and Quality Control

Raw snRNA-seq data in 10× Genomics HDF5 format were processed in a Python environment using the Scanpy 1.12.1 library [18,19]. Quality control was performed independently for each sample. Nuclei were retained based on the following criteria: 200–4000 detected genes, at least 500 unique molecular identifiers (UMIs), maximum 10% mitochondrial reads, and maximum 5% erythrocyte reads. Genes detected in fewer than three nuclei were excluded from the dataset.

#### 2.6.2. Doublet Detection

A dual-strategy approach was employed to remove doublets. Algorithmic detection was performed per sample using DoubletFinder [20], which was re-implemented by the authors from R to Python as pydoubletfinder [21] (10 principal components, pN = 0.25, pK = 0.09, dynamic doublet rate base of 3%). To address high-ambient RNA levels in heart tissue, this was supplemented with biological signature-based filtering. Clusters and individual nuclei showing high expression of curated signature genes indicative of non-target cell contamination (e.g., cardiomyocytes, fibroblasts, endothelial cells) were removed.

#### 2.6.3. Normalization, Integration, and Global Clustering

Raw UMI counts were normalized to 10,000 reads per nucleus and log-transformed. The top 5000 highly variable genes were selected using the Seurat v3 method [22]. To account for technical batch effects, data integration was executed using the multi-resolution Variational Inference (mrVI) model [23] with 30 latent dimensions, a maximum of 400 epochs, and a batch size of 4096. A k-nearest neighbours graph (k = 30) was constructed on the latent representation, followed by Leiden clustering at a resolution selected by an automated two-stage grid search (coarse scan: nine values from 0.1 to 2.0; fine scan: step 0.05 around the coarse optimum), using the criterion of maximizing the number of distinct annotatable cell type labels recovered. The final resolution was 2.10 for the global compartment (42 clusters; modularity 0.818) and 2.05 for the macrophage subcluster (26 clusters; modularity 0.828), with n_iterations = 2 and random_state = 42 [24]. Major cardiac cell types were annotated using a gene signature scoring method combined with majority voting per cluster.

#### 2.6.4. Macrophage Subclustering and Annotation

Cells annotated as macrophages were isolated, filtered for residual doublets, and re-integrated using mrVI with 20 latent dimensions. Following graph construction (k = 15) and Leiden clustering, cells were classified into five functional and ontogenetic subpopulations. Annotation utilized a differential expression overlap strategy, where the top 50 differentially expressed genes per cluster (Wilcoxon test) were compared against curated subpopulation markers. Final annotations were refined using logic rules based on temporal disease dynamics (e.g., differentiating embryonic-derived resident macrophages from monocyte-derived resident-like cells based on their presence at day 0) and the expression frequencies of specific functional markers within the clusters. The boundary between Mac-MHCII and Classical Monocytes (Mono-C) was not defined by the shared MHC-II machinery (*Cd74*, *H2-Eb1*, *Ciita*), which is co-expressed by both populations, but by lineage-defining markers: Mono-C identity required the co-expression of the classical-monocyte programme (*Ccr2*, *Ly6c2*, *Plac8*, *Sell*, *Vcan*), whereas Mac-MHCII required positivity for maturation markers (Mertk, Cx3cr1), together with absence of that programme, irrespective of MHC-II level (Supplementary Figure S1 and Supplementary Table S4).

#### 2.6.5. Differential Gene Expression (DGE) and Enrichment Analysis

DGE analysis was conducted using a pseudobulk approach to mitigate Type I error inflation [25]. UMI counts were aggregated by sample and cell type. Statistical testing was performed using PyDESeq2 [26] with a design formula incorporating the timepoint. Data were filtered to require a minimum of 10 cells per sample, at least 50 aggregated UMIs per gene, and a minimum of three replicates per group. Significance was established at an adjusted *p*-value (FDR) below 0.05 and an absolute log2 fold change of at least 1.0. Gene Set Enrichment Analysis (GSEA) was performed using GSEApy [27] in prerank mode against the GO Biological Process 2021 database [28]. Pathways with an adjusted *p*-value below 0.25 were considered significantly enriched.

#### 2.6.6. Cell–Cell Communication

Ligand–receptor interactions were inferred per timepoint using the LIANA library’s rank aggregate method and the mouse consensus database [29]. Analysis required a minimum of five cells per type. Significant interactions were defined by an adjusted rank score of 0.1 or lower.

Cell types represented by fewer than five nuclei at a given timepoint were excluded from the interaction analysis at that timepoint to avoid computing ligand–receptor scores from unstable, single-digit expression estimates. At day 0, only neutrophils (zero nuclei) and adipocytes (four nuclei) fell below this threshold, and neither contributes to the macrophage–stromal axes that form the focus of this study. Four further populations passing the threshold (T cells, resident macrophages, arterial endothelial cells and B cells; five to eight nuclei) yielded no significant interactions at day 0, which is an outcome insensitive to the threshold value. The lower interaction count at day 0 therefore reflects the genuine transcriptional quiescence of the healthy myocardial communication network rather than a filtering artefact (Supplementary Table S2).

#### 2.6.7. Reproducibility

A fixed random seed (42) was applied to all stochastic analysis steps (Leiden clustering, UMAP, mrVI, DoubletFinder) to ensure deterministic results. The analysis pipeline and configuration files are available from the corresponding author upon reasonable request. The pydoubletfinder re-implementation, together with a Docker environment that reproduces the full benchmarking against the original R DoubletFinder [20] reported in Supplementary Table S3, is publicly available at https://github.com/dam2452/doubletfinder-py, accessed on 4 May 2026.

## 3. Results

**Figure 1:**
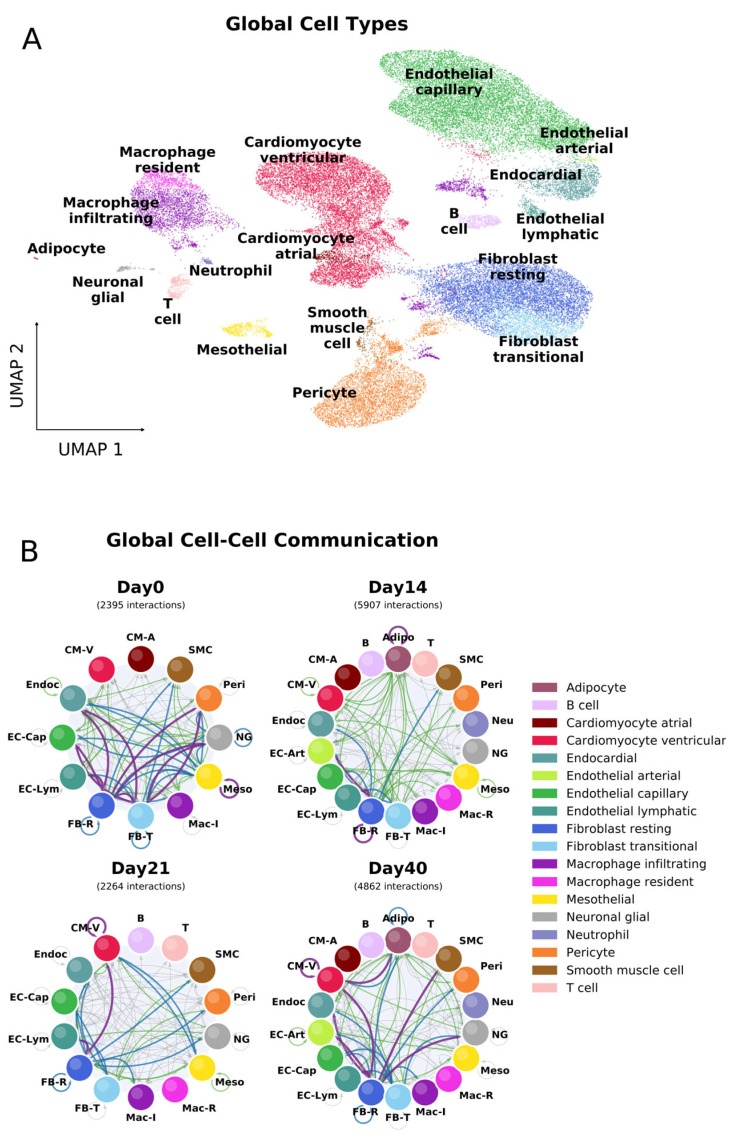
Global cell type composition and cell–cell communication dynamics in the cardiac tissue during experimental autoimmune myocarditis progression. (**A**) UMAP projection of all profiled nuclei. A total of 18 transcriptionally distinct cell populations was identified, encompassing cardiomyocyte subpopulations (ventricular, CM-V; atrial, CM-A), stromal cells (fibroblast resting, FB-R; fibroblast transitional, FB-T; pericyte, Peri; smooth muscle cell, SMC; mesothelial, Meso), endothelial subtypes (capillary, EC-Cap; arterial, EC-Art; lymphatic, EC-Lym; endocardial, Endoc), immune cells (macrophage resident, Mac-R; macrophage infiltrating, Mac-I; T cell, T; neutrophil, Neu; B cell, B), and minor populations (adipocyte, Adipo; neuronal/glial, NG). (**B**) Chord diagrams depicting global cell–cell communication networks inferred at day 0 (2395 interactions), day 14 (5907 interactions), day 21 (2264 interactions), and day 40 (4862 interactions). Each node represents a cell type, coloured according to the legend. Edge width reflects the number of ligand–receptor interactions between each pair of cell types. Abbreviations as in (**A**).

To characterize the cellular composition of the cardiac tissue and the dynamics of intercellular communication across experimental autoimmune myocarditis (EAM) progression, we performed single-nuclei RNA-sequencing of four timepoints during disease progression and visualized the data using UMAP dimensionality reduction. Our analysis of 46,233 nuclei revealed the presence of 18 transcriptionally distinct cell populations (Figure 1A), including two cardiomyocyte subpopulations, namely ventricular (CM-V) and atrial (CM-A), which formed large, densely populated clusters at the centre of the embedding. The stromal compartment contained fibroblast resting and fibroblast transitional subsets, pericytes, smooth muscle cells, and mesothelial cells, while the endothelial lineage was composed of four subtypes, namely capillary, arterial, lymphatic, and endocardial. Immune cell populations, including resident and infiltrating macrophages, T cells, neutrophils, and B cells, occupied distinct peripheral regions of the UMAP, with adipocytes and neuronal/glial cells identified as minor populations.

To assess the extent of intercellular crosstalk over time, we inferred ligand–receptor interaction networks at four timepoints (Figure 1B). The total number of predicted interactions varied substantially across the time course, with 2395 interactions detected at day 0, rising to a peak of 5907 at day 14, followed by 2264 at day 21, and 4862 at day 40. This pattern suggests a pronounced amplification of intercellular signalling during the inflammatory phase at day 14, with fibroblast subpopulations, macrophages, and endothelial cells serving as major communication hubs. The subsequent decline at day 21 and partial rebound at day 40 likely reflect a transition from inflammatory signalling towards a remodelling phase, in which macrophages and fibroblasts consistently maintained central roles in coordinating the cellular response. Among the most prominent macrophage–fibroblast interactions at day 14 were the SPP1–CD44, POSTN–ITGAV, and CCL2–CCR2 ligand–receptor pairs, suggesting roles in fibroblast activation and monocyte recruitment (the complete inferred ligand–receptor interaction set for all timepoints is provided in Supplementary Table S2).

**Figure 2:**
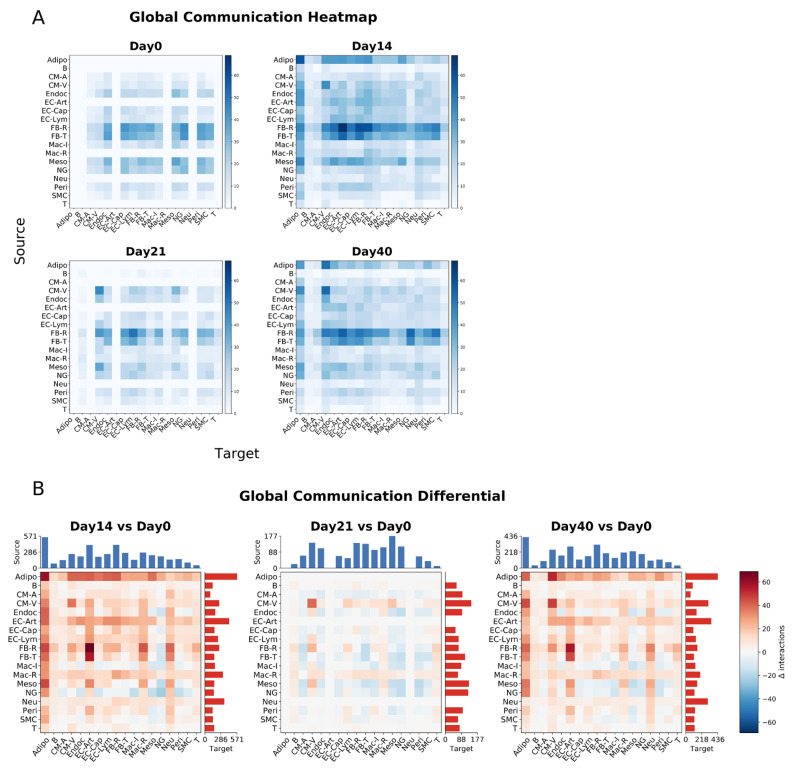
Quantification of global cell–cell communication patterns and their temporal dynamics. (**A**) Heatmaps depicting the number of inferred ligand–receptor interactions between all cell type pairs at day 0, day 14, day 21, and day 40. Rows represent source (sending) cell types and columns represent target (receiving) cell types. Colour intensity reflects the total number of interactions, ranging from low (white) to high (dark blue). (**B**) Differential communication heatmaps comparing interaction counts at day 14, day 21, and day 40 relative to day 0. Red indicates a gain and blue indicates a loss in the number of interactions compared to baseline. Bar plots flanking each heatmap represent the aggregated differential interaction counts per source (**top**) and target (**right**) cell type. Abbreviations as in Figure 1.

To quantify the directionality and magnitude of intercellular signalling between all cell type pairs, we generated global communication heatmaps for each timepoint (Figure 2A). At day 0, interaction counts were broadly distributed across most cell type pairs, with two fibroblast subpopulations (resting FB-R and transitional FB-T) and macrophages (Infiltrating Mac-I and resident Mac-R) displaying relatively elevated signalling activity both as sources and targets. At day 14, a marked global increase in communication intensity was observed, with FB-R emerging as the dominant signalling source, exhibiting particularly strong interactions with multiple target cell types. This pattern partially persisted at day 40, whereas day 21 showed an overall reduction in communication intensity across the majority of cell type pairs, consistent with the interaction counts reported in Figure 1B.

To further delineate timepoint-specific changes relative to baseline, we computed differential communication maps, comparing each post-baseline timepoint to day 0 (Figure 2B). The day 14 vs. day 0 comparison revealed a broad and pronounced gain in interactions, with the majority of cell type pairs showing increased signalling (red), particularly involving FB-R as a source and multiple endothelial and stromal cell types as targets. The FB-R to FB-T interaction pair exhibited the largest absolute increase among all pairs. In contrast, the day 21 vs. day 0 comparison showed a more heterogeneous pattern, with modest gains in some pairs and losses in others, suggesting a partial normalization of the signalling landscape. The day 40 vs. day 0 comparison revealed a renewed but more selective increase in interactions, predominantly involving fibroblast and endothelial populations, indicative of ongoing tissue remodelling processes at the later disease stage.

**Figure 3:**
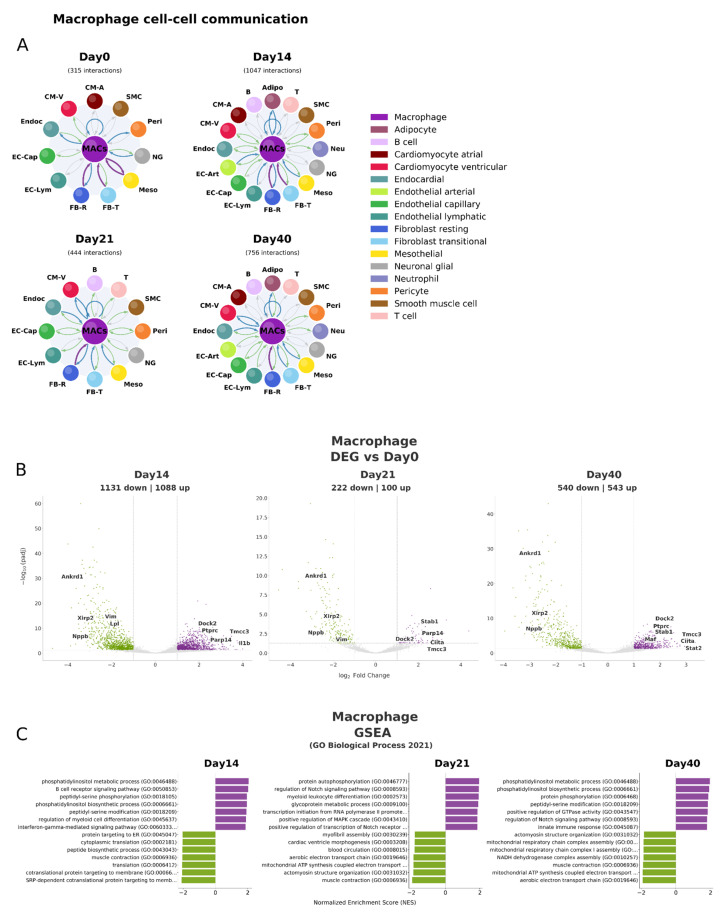
Macrophage-centred cell–cell communication, differential gene expression, and pathway enrichment across disease timepoints. (**A**) Chord diagrams depicting cell–cell communication networks centred on macrophages (MACs) at day 0 (315 interactions), day 14 (1047 interactions), day 21 (444 interactions), and day 40 (756 interactions). Edge width reflects the number of ligand–receptor interactions between each pair of cell types. The macrophage node is highlighted at the centre of each diagram. (**B**) Volcano plots showing differentially expressed genes (DEGs) in macrophages at day 14, day 21, and day 40 relative to day 0. The x-axis represents log_2_ fold change, and the y-axis represents −log_10_-adjusted *p*-value. Downregulated and upregulated gene counts are indicated above each plot. Selected genes of interest are labelled. (**C**) Bar plots of Gene Set Enrichment Analysis (GSEA) results based on GO Biological Process 2021 gene sets for macrophages at each timepoint relative to day 0. Bars extending to the right (green) indicate positively enriched pathways and bars extending to the left (purple) indicate negatively enriched pathways. The x-axis represents the Normalized Enrichment Score (NES). Abbreviations as in Figure 1.

To investigate the role of macrophages as key mediators of cardiac intercellular signalling, we focused on macrophage-centred cell–cell communication networks across all timepoints (Figure 3A). At day 0, macrophages participated in 315 interactions, engaging predominantly with fibroblast subpopulations, cardiomyocytes, and endothelial cells. At day 14, macrophage-associated interactions expanded dramatically to 1047, with macrophages acting as central hubs communicating with virtually all cell types present in the tissue, including newly appearing populations such as adipocytes, B cells, and neutrophils. This broad communicative activity subsided at day 21 (444 interactions) and day 40 (756 interactions), though macrophages retained their hub-like connectivity at both later timepoints, maintaining particularly prominent interactions with fibroblast and endothelial compartments.

To characterize the transcriptional states underlying these communication dynamics, we performed differential gene expression analysis in macrophages at each timepoint relative to day 0 (Figure 3B). At day 14, the most extensive transcriptional remodelling was observed, with 1131 genes downregulated and 1088 upregulated. Among the most significantly changed genes, *Dock2*, *Ptprc*, *Parp14*, and *Tmcc3* were notably upregulated, while *Ankrd1*, *Xirp2*, *Vim*, *Lpl*, and *Nppb* were among the downregulated genes. At day 21, the transcriptional response was considerably more modest (222 down, 100 up), with *Stab1*, *Dock2*, *Parp14*, *Ciita*, and *Tmcc3* among the upregulated genes. At day 40, a renewed transcriptional shift was observed (540 down, 543 up), with *Dock2*, *Ptprc*, *Maf*, *Ciita*, *Stat2*, and *Tmcc3* upregulated, suggesting sustained but evolving macrophage activation at the later disease stage (Figure 3B).

Gene Set Enrichment Analysis (GSEA) of macrophage transcriptomes revealed distinct pathway enrichment profiles at each timepoint (Figure 3C). At day 14, upregulated pathways included B cell receptor signalling, interferon-gamma-mediated signalling, and the regulation of myeloid cell differentiation, while downregulated pathways were associated with muscle contraction, translation, and translational protein targeting, reflecting a shift away from homeostatic functions toward immune activation. At day 21, enriched pathways included the regulation of Notch signalling, myeloid leukocyte differentiation, the positive regulation of MAPK cascade, and cardiac ventricle morphogenesis, alongside the suppression of actomyosin organization and muscle contraction-related processes. At day 40, upregulated pathways encompassed phosphatidylinositol metabolic processes, protein phosphorylation, innate immune response, and Notch signalling regulation, while mitochondrial respiratory chain complex assembly and aerobic electron transport chain pathways were among those negatively enriched, suggesting metabolic reprogramming alongside sustained immune activity in macrophages at this stage (Figure 3C).

**Figure 4:**
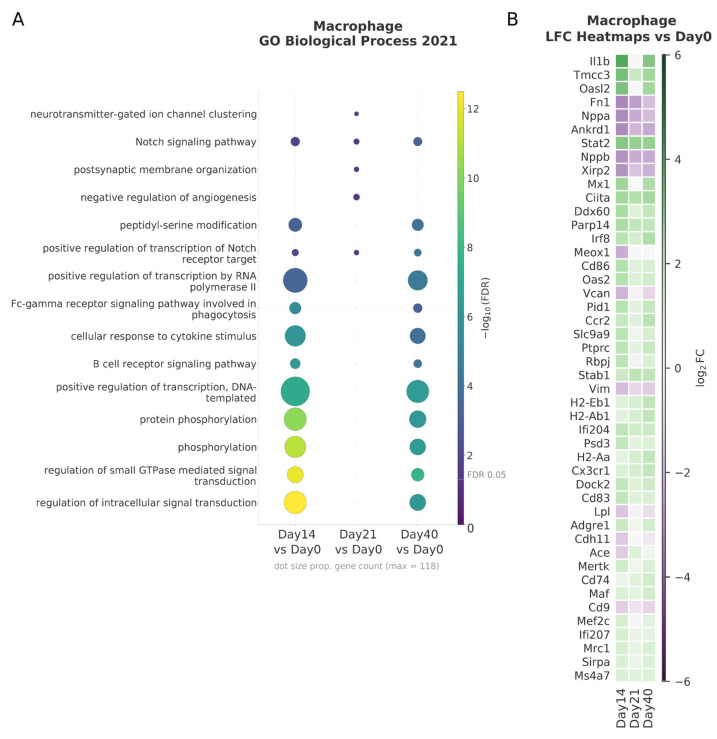
GO Biological Process enrichment and gene-level log_2_ fold change dynamics in macrophages across disease timepoints. (**A**) Dot plot of GO Biological Process 2021 enrichment results for macrophages at day 14, day 21, and day 40 relative to day 0. Each dot represents a significantly enriched biological process (FDR < 0.05). Dot size is proportional to the number of genes contributing to the enrichment (maximum = 118), and dot colour reflects the −log_10_(FDR), ranging from low (purple) to high (yellow) significance. Only selected pathways enriched in at least one comparison are shown. (**B**) Heatmap of log_2_ fold change (LFC) values for selected macrophage genes at day 14, day 21, and day 40 relative to day 0. Green indicates upregulation and purple indicates downregulation. Genes were selected based on their biological relevance to macrophage identity, activation, and tissue remodelling. Abbreviations as in Figure 1.

To further resolve the biological processes underlying macrophage transcriptional reprogramming, we performed GO Biological Process enrichment analysis and visualized the results across all three comparisons, i.e., day 14 vs. day 0, day 21 vs. day 0 and day 40 vs. day 0 (Figure 4A). Several pathways were consistently enriched across multiple timepoints, indicating sustained transcriptional alterations in macrophages throughout disease progression. Notably, positive regulation of transcription (DNA-templated and RNA polymerase II-dependent), protein phosphorylation, phosphorylation, and regulation of intracellular signal transduction were significantly enriched at both day 14 and day 40, with the largest gene counts and highest significance scores observed at day 14. The Notch signalling pathway and positive regulation of transcription of Notch receptor targets were recurrently enriched across all three timepoints, underscoring the sustained activation of this pathway in macrophages. Fc-gamma receptor signalling involved in phagocytosis, cellular response to cytokine stimulus, and B cell receptor signalling pathway were prominently enriched at day 14, with attenuated but detectable enrichment at day 40. At day 21, enrichment was generally less pronounced, with only a limited number of pathways reaching significance, consistent with the reduced transcriptional activity observed at this intermediate timepoint (Figure 4A).

To visualize the directionality and consistency of individual gene expression changes across timepoints, we generated a log_2_ fold change heatmap for a curated set of macrophage marker and effector genes (Figure 4B). Genes such as *Il1b*, *Tmcc3*, *Oasl2*, *Stat2*, *Mx1*, *Ciita*, *Ddx60*, *Parp14*, and *Irf8* showed persistent upregulation across all three timepoints, suggesting their involvement in a sustained macrophage activation state. Conversely, a distinct subset of genes, including *Fn1*, *Ankrd1*, *Vcan*, *Vim*, *Lpl*, and *Cd9*, was consistently downregulated throughout the disease course relative to day 0. In contrast, genes associated with tissue-resident macrophage identity and homeostatic function, including *Cx3cr1*, *Mrc1*, *Sirpa*, *Ms4a7*, *Mertk*, *Cd74*, and *Maf*, maintained mildly positive expression, indicating a preservation of the resident macrophage transcriptional signature over the course of disease. Several genes displayed timepoint-specific patterns: *Lpl* and *Vim* were most strongly downregulated at day 14, while *Irf8*, *Parp14*, *Ciita*, and *Ddx60* showed more selective upregulation at day 14 and day 40. Together, these findings support a model in which macrophages undergo a dynamic and partially reversible shift from a homeostatic resident phenotype toward a pro-inflammatory and transcriptionally activated state, with distinct waves of gene expression remodelling at inflammatory and later disease stages (Figure 4B).

**Figure 5:**
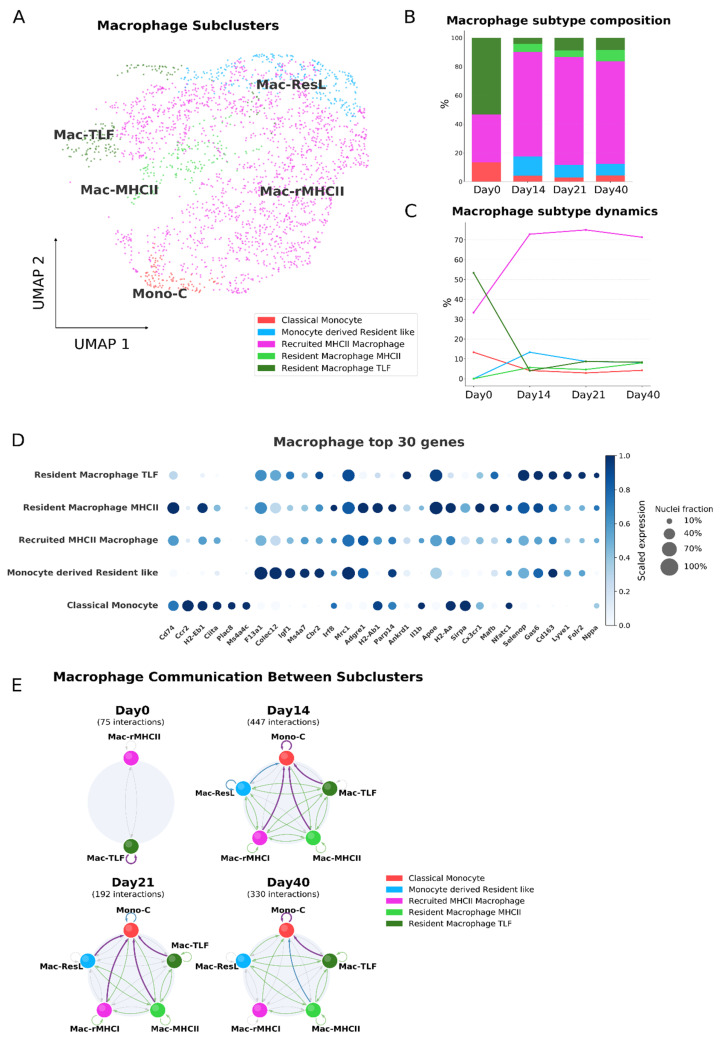
Macrophage subclustering, compositional dynamics, marker gene expression, and intra-macrophage communication. (**A**) UMAP projection of macrophage subclusters, coloured by subpopulation identity. Five distinct subpopulations were identified: resident macrophage TLF (Mac-TLF), resident macrophage MHCII (Mac-MHCII), recruited MHCII macrophages (Mac-rMHCII), monocyte-derived resident-like macrophages (Mac-ResL), and classical monocyte (Mono-C). Among these, Mac-ResL emerged exclusively at the subclustering level, drawing cells from both Mac-R and Mac-I initial clusters, consistent with its transitional identity at the interface of recruited monocytes and resident macrophages and its transcriptional similarity to both parent compartments at lower resolution. (**B**) Stacked bar plots showing the proportional composition of macrophage subpopulations at each timepoint. (**C**) Line plots depicting the temporal dynamics of each macrophage subtype as a percentage of the total macrophage pool across day 0, day 14, day 21, and day 40. (**D**) Dot plot of the top 30 marker genes across macrophage subpopulations. Dot size represents the fraction of nuclei expressing each gene and dot colour reflects scaled expression level. (**E**) Chord diagrams depicting cell–cell communication networks among macrophage subpopulations at day 0 (75 interactions), day 14 (447 interactions), day 21 (192 interactions), and day 40 (330 interactions). Edge width reflects the number of ligand–receptor interactions between each pair of cell types. Abbreviations as in Figure 1.

To dissect macrophage heterogeneity at higher resolution, we performed subclustering of the macrophage compartment, identifying five transcriptionally distinct subpopulations (Figure 5A): resident macrophage TLF (Mac-TLF), resident macrophage MHCII (Mac-MHCII), recruited MHCII macrophages (Mac-rMHCII), monocyte-derived resident-like macrophages (Mac-ResL), and classical monocytes (Mono-C).

Analysis of subtype composition across timepoints revealed pronounced shifts in the macrophage compartment during disease progression (Figure 5B,C). At day 0, the macrophage pool was dominated by tissue-resident subpopulations, with Mac-TLF (CCR2-) representing the largest fraction (approximately 55%), followed by Mac-MHCII. Recruited subpopulations (Mac-rMHCII, Mono-C) and the transitional Mac-ResL population together constituted only a minor proportion of the compartment at baseline. At day 14, a dramatic compositional shift occurred, with recruited MHCII macrophages (Mac-rMHCII) expanding to account for over 70% of all macrophages, while the resident Mac-TLF population contracted sharply. This dominance of the recruited macrophage subtype was maintained at day 21 and day 40, where Mac-rMHCII continued to constitute the majority of the macrophage compartment, indicating a sustained and largely irreversible infiltration of recruited macrophages following the initial inflammatory stimulus.

Subtype-specific gene expression profiles were characterized using a dot plot of the top 30 marker genes across all five macrophage subpopulations (Figure 5D). Classical monocytes (Mono-C) were distinguished by the high expression of *Ccr2*, *Plac8*, and *Ms4a4c*, together with MHC class II-related genes (*Cd74*, *H2-Eb1*, *Ciita*), reflecting an inflammatory Ly6C^hi^ monocyte identity with concurrent antigen-presentation capacity. Resident macrophage MHCII (Mac-MHCII) showed the strongest expression of the antigen-presentation programme (*Cd74*, *H2-Eb1*, *H2-Ab1*, *H2-Aa*, *Ciita*) combined with the tissue-resident transcription factor *Mafb*, while lacking *Ccr2*. Recruited MHCII macrophages (Mac-rMHCII) expressed *Ankrd1*, *Parp14*, *Il1b*, and *Apoe*, consistent with an activated, inflammation-associated phenotype. Mac-TLF cells showed enrichment for the canonical resident signature (*Lyve1*, *Folr2*, *Cd163*) together with *Mafb*, *Cx3cr1*, *Selenop*, *Gas6*, and *Mrc1*. Monocyte-derived resident-like macrophages (Mac-ResL) displayed a transitional profile, with the high expression of *F13a1*, *Colec12*, *Igf1*, and *Ms4a7* alongside both monocyte- and resident-associated markers, suggesting an intermediate differentiation state. The complete list of marker genes for each subpopulation is provided in Supplementary Table S1.

To assess whether macrophage subclusters engage in intercellular communication among themselves, we analyzed intra-macrophage subcluster interaction networks across timepoints (Figure 5E). At day 0, only 75 interactions were detected, with limited connectivity predominantly involving Mac-rMHCII and Mac-TLF. At day 14, intra-macrophage communication expanded markedly to 447 interactions, with all five subpopulations engaged in dense bidirectional signalling, and classical monocytes and Mac-TLF emerging as prominent interaction hubs. At day 21 (192 interactions) and day 40 (330 interactions), communication networks remained more complex than at baseline, with Mac-ResL, Mac-MHCII, and Mac-rMHCII maintaining substantial connectivity, suggesting ongoing intercellular coordination within the macrophage compartment during the remodelling phase.

**Figure 6:**
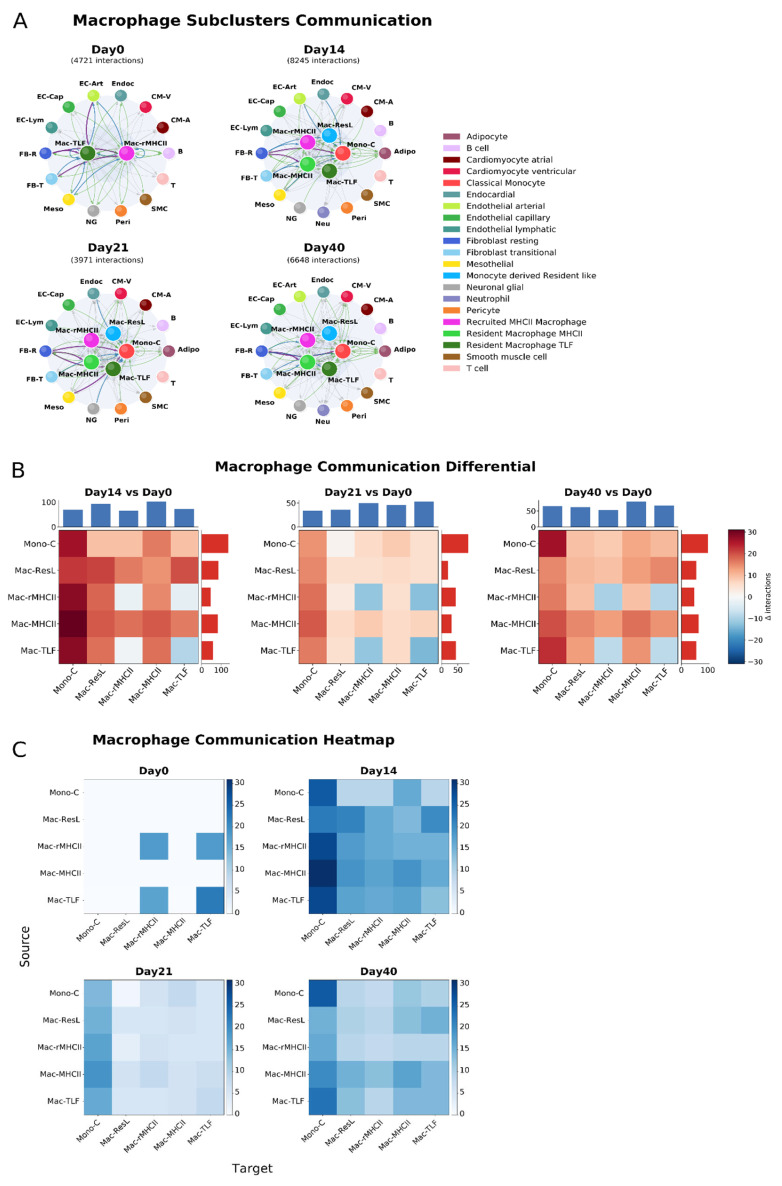
Macrophage subcluster-resolved cell–cell communication networks, differential interactions, and absolute interaction heatmaps across disease timepoints. (**A**) Chord diagrams depicting cell–cell communication networks with macrophage subclusters resolved at day 0 (4721 interactions), day 14 (8245 interactions), day 21 (3971 interactions), and day 40 (6648 interactions). Edge width reflects the number of ligand–receptor interactions between each pair of cell types. Macrophage subpopulations are represented by distinct colours as indicated in the legend. (**B**) Differential communication heatmaps comparing macrophage subcluster interaction counts at day 14, day 21, and day 40 relative to day 0. Red indicates a gain and blue indicates a loss in the number of interactions. Bar plots represent aggregated differential interaction counts per source (top) and target (right) subpopulation. (**C**) Heatmaps depicting absolute numbers of inferred ligand–receptor interactions between macrophage subpopulations at each timepoint. Rows represent source and columns represent target subpopulations. Colour intensity reflects the total number of interactions, ranging from low (white) to high (dark blue). Abbreviations as in Figure 1 and Figure 5.

To examine how individual macrophage subpopulations contribute to tissue-wide intercellular communication, we resolved cell–cell interaction networks at the subcluster level across all timepoints (Figure 6A). Because each macrophage subpopulation was treated as a separate communicating node, the total interaction counts at this resolution exceed those reported in the global analysis (Figure 1B), where all macrophages were collapsed into a single entity—additional interactions become detectable both between macrophage subclusters themselves and between individual subclusters and other cardiac cell types (Figure 6A).

At day 0, a total of 4721 interactions was detected, with Mac-rMHCII and Mac-TLF serving as the dominant signalling nodes, maintaining extensive connections with fibroblast subpopulations and endothelial cells. At day 14, the total number of interactions increased substantially to 8245, with all macrophage subpopulations showing expanded connectivity. Classical monocytes (Mono-C) and Mac-ResL emerged as prominent hubs at this timepoint, alongside the already active Mac-rMHCII and Mac-TLF populations. At day 21, interactions decreased to 3971, and the network structure became less dense, though Mac-TLF and Mono-C retained notable signalling activity. At day 40, interactions recovered to 6648, with a broadly distributed communication pattern involving all macrophage subtypes and multiple non-macrophage cell types, particularly fibroblasts and endothelial cells.

Differential communication analysis between macrophage subpopulations relative to day 0 revealed subtype-specific and timepoint-specific changes in signalling (Figure 6B). At day 14, the most pronounced gains in interactions were observed for Mono-C as both a source and a target, with large positive differentials across all receiving subpopulations. Mac-ResL also showed a substantial increase in outgoing interactions. Mac-TLF similarly exhibited broad interaction gains across the network. At day 21, the differential landscape was more modest overall, with Mono-C retaining elevated interaction gains while Mac-rMHCII showed a relative reduction in communication compared to baseline. At day 40, Mono-C again displayed the largest positive differential as a source, particularly in interactions directed toward Mac-ResL and Mac-rMHCII, while Mac-rMHCII showed net decreases in several target pairs, suggesting a redistribution of signalling roles within the macrophage compartment at the later disease stage.

Absolute interaction counts between macrophage subpopulations confirmed these dynamics (Figure 6C). At day 0, interactions were sparse and largely restricted to Mac-rMHCII and Mac-TLF pairs. At day 14, a global increase in communication intensity was evident across virtually all subcluster pairs, with Mono-C emerging as both a major sender and receiver. At day 21 and day 40, interaction counts remained elevated relative to day 0, with Mono-C maintaining high communication activity and Mac-ResL showing sustained increase in its interaction strength over time, consistent with its proposed role as a transitional state bridging recruited monocytes and resident macrophage identity.

## 4. Discussion

The present study provides a comprehensive view of the dynamic changes in cardiac macrophage populations during the progression of experimental autoimmune myocarditis (EAM) to late cardiac remodelling, highlighting their unique functional specialization. Our findings support and extend previous observations that myocarditis is not only a transient inflammatory condition but a highly orchestrated process involving temporally regulated immune–stromal interactions that critically determine the outcome of the disease [9,30].

Consistent with earlier reports [8,9], we observed a rapid depletion of tissue-resident macrophages (TRMs, CCR2^−^, TLF^+^) during the early inflammatory phase of EAM. This loss was accompanied by a pronounced influx of CCR2^+^ monocyte-derived macrophages, which dominated the immune landscape at peak inflammation. Indeed, subclustering of the macrophage compartment revealed five transcriptionally distinct subpopulations: resident macrophage TLF (Mac-TLF), resident macrophage MHCII (Mac-MHCII), recruited MHCII macrophages (Mac-rMHCII), monocyte-derived resident-like macrophages (Mac-ResL), and classical monocytes (Mono-C) whose proportions shifted dramatically across the disease course. At day 0, Mac-TLF represented the largest fraction of the macrophage pool (~55%), whereas by day 14 the recruited MHCII macrophage subpopulation (Mac-rMHCII) expanded to constitute over 70% of all macrophages, a dominance that was maintained at day 21 and day 40. Single-nuclei transcriptomic profiling revealed that these recruited macrophages exhibit a strongly pro-inflammatory signature characterized by the elevated expression of *Parp14*, *Il1b*, *Apoe* and *Stat2*. Notably, the expansion of Mac-rMHCII was one of the most prominent features of the inflammatory phase, suggesting its central role in amplifying inflammatory signalling within the myocardium.

Importantly, our temporal analysis indicates that macrophage heterogeneity increases as the disease progresses. While early-stage macrophages are predominantly pro-inflammatory, later timepoints, particularly day 21 and day 40, reveal the emergence of distinct subsets with mixed or reparative phenotypes, including the monocyte-derived resident-like (Mac-ResL) population, which displayed a transitional gene expression profile combining monocyte-associated and resident macrophage markers, suggesting an intermediate differentiation state. Genes associated with tissue remodelling and resolution, including *Mrc1*, *Maf*, *Folr2*, and *Cx3cr1*, were persistently expressed in resident-like populations at later timepoints. However, this reparative response appears insufficient to fully restore homeostasis, likely due to the persistence of inflammatory niches that sustain fibroblast activation and extracellular matrix deposition [31].

A further limitation of the present study is the exclusive use of male BALB/c mice. This was a deliberate experimental decision, as male mice develop more consistent and severe EAM than females, exhibiting stronger pro-inflammatory macrophage responses, greater cardiac infiltration, and more pronounced fibrotic remodelling, which are features that are well-documented in both autoimmune and viral myocarditis models and align with the higher incidence and severity of myocarditis observed in men [32,33,34]. Nevertheless, this choice limits the generalizability of our findings. Sex hormones are known to modulate macrophage polarization, with estradiol attenuating pro-inflammatory cytokine production, including IL-1β, and testosterone promoting M1 polarization and cardiac remodelling [35]. Whether the macrophage subpopulation dynamics and immune–stromal interactions described here are recapitulated or modified in female mice therefore remains an open question that warrants investigation in future studies.

It should be noted that ligand–receptor interaction analysis is inherently hypothesis-generating, and the inferred macrophage–fibroblast interactions identified here require experimental validation to establish functional significance. Our data supports the concept that macrophage function is tightly linked to their intercellular communication context, as previously described [14]. By integrating transcriptional signatures with cell–cell interaction analysis, we identified discrete inflammatory microenvironments characterized by close interactions between macrophages and fibroblasts. These interactions were most pronounced at day 14, when macrophage-centred communication networks reached their peak (1047 interactions), with fibroblast subpopulations (FB-R, FB-T) consistently among the primary targets of macrophage signalling across all timepoints. Furthermore, differential communication analysis revealed that fibroblast resting cells (FB-R) emerged as the dominant signalling source at day 14, exhibiting the largest absolute increase in interactions compared to day 0, particularly in exchanges directed toward endothelial and other stromal cell types. In line with recent spatial transcriptomics studies [14], we observed that fibroblasts within these regions adopt a pro-inflammatory phenotype, marked by increased expression of *Ccl2*, *Ccl7*, and *Csf2*, further reinforcing local immune cell accumulation. These findings provide a precise cellular context for the TGF-β-dependent cytokine regulation observed in our previous work [6]. They suggest that while TGF-β signalling modulates the secretory profile of Mac-rMHCII, the progression of fibrosis is more likely driven by intense macrophage–fibroblast crosstalk rather than macrophage-intrinsic TGF-β pathways.

Furthermore, our results highlight the critical role of macrophage-derived cytokines in shaping fibroblast behaviour. Elevated expression of *Il1b*, primarily driven by the classical monocyte (Mono-C) subpopulation, correlated with increased activation of pro-fibrotic pathways in fibroblasts, including the upregulation of collagen genes (*Col1a1*, *Col3a1*) and extracellular matrix regulators. This is consistent with previous findings demonstrating that IL-17A-driven inflammation synergizes with macrophage-derived signals to promote maladaptive remodelling [10,13].

In addition to the role of macrophages in inflammation and fibrosis, our data suggest that disrupted macrophage–cardiomyocyte interactions may contribute to functional decline. The GSEA of macrophage transcriptomes at day 14 revealed significant negative enrichment of pathways related to translation and muscle contraction. This aligns with previous reports indicating that impaired macrophage-mediated clearance of damaged mitochondria can exacerbate cardiomyocyte dysfunction [12].

From a translational perspective, our findings open new avenues for precision immunomodulatory therapies. The coexistence of pro-inflammatory (Mac-rMHCII, Mono-C) and potentially reparative (Mac-ResL, Mac-TLF) macrophage subsets suggests that broad immunosuppression may unintentionally impair resolution processes [9,10,30]. Instead, our data support the development of targeted strategies aimed at selectively modulating specific macrophage populations or their interactions with fibroblasts [14]. Whether the inflammatory imprint established by sustained Mac-rMHCII dominance is reversible, and to what extent it conditions subsequent ventricular remodelling, remains an open question. In this context, the framework proposed by [36], which delineates the conditions under which cardiac function recovers in reversible and inflammation-associated left ventricular dysfunction, provides a valuable clinical reference for interpreting the macrophage-driven LV dysfunction observed here and for defining the therapeutic window within which precision immunomodulation may preserve myocardial viability.

Nevertheless, this study has several limitations. While snRNA-sequencing provides high-resolution insight into transcriptional alterations, it does not fully capture protein-level activity or functional outcomes. Furthermore, although the day 40 timepoint corresponds to an established stage of the EAM model, the absence of echocardiographic assessment of ventricular dimensions and function, as well as formal fibrosis quantification, precludes a definitive designation of dilated cardiomyopathy. This stage is therefore described as late remodelling. Additionally, the lack of direct spatial validation limits our ability to definitively map cellular interactions in situ. Future studies integrating spatial transcriptomics, proteomics, and functional assays will be essential to validate and extend these findings.

The translational relevance of murine EAM models to human inflammatory heart disease warrants careful consideration. The α-MyHC-induced EAM model recapitulates key features of human myocarditis, including T cell-driven inflammation, macrophage infiltration, and progression to adverse cardiac remodelling, making it a well-established platform for mechanistic studies [37]. Nevertheless, important differences between murine and human cardiac immunology must be acknowledged. Species-specific differences in macrophage ontogeny, cytokine networks, and the composition of the cardiac immune niche may limit direct extrapolation of findings [7]. Furthermore, human myocarditis is aetiologically heterogeneous, encompassing viral, autoimmune, and immune checkpoint inhibitor-associated forms, each potentially engaging distinct macrophage programmes [2].

Despite these caveats, the macrophage subpopulations and signalling axes identified here, particularly CCR2-mediated monocyte recruitment, the SPP1–CD44 axis, and macrophage–fibroblast crosstalk, have meaningful correlates in human cardiac pathology. CCR2^+^ macrophages have been identified in human failing hearts and associated with adverse remodelling [9], while SPP1-expressing macrophages have emerged as a conserved pro-fibrotic population across human inflammatory cardiomyopathies [30]. While direct therapeutic breakthroughs cannot be claimed on the basis of transcriptomic data alone, the framework established here provides a rational basis for the design of precision immunomodulatory interventions, the efficacy of which will ultimately require validation in human cohorts and clinical trials.

The potential impact of corticosteroid therapy on the macrophage dynamics described in this study merits consideration, given that steroids represent the mainstay of immunosuppressive treatment in certain forms of myocarditis and inflammatory cardiomyopathy [38,39]. Corticosteroids exert broad immunomodulatory effects, including the suppression of pro-inflammatory cytokine production and the promotion of macrophage differentiation toward anti-inflammatory phenotypes with an enhanced capacity to suppress T cell activation [40]. In the context of our findings, steroid therapy would be expected to attenuate the dramatic expansion of CCR2^+^ Mac-rMHCII macrophages observed at day 14, reduce macrophage-derived *Il1b* and *Stat2* signalling, and dampen the pro-inflammatory macrophage–fibroblast crosstalk mediated through CCL2, CCL7, and SPP1 axes. However, such broad-spectrum suppression could impair reparative macrophage subsets like Mac-TLF and Mac-ResL, thereby disrupting their contributions to cardiac homeostasis and the resolution of injury [9]. This non-selective suppression may partly explain why steroid therapy does not uniformly improve outcomes across all forms of myocarditis—with benefit demonstrated primarily in virus-negative inflammatory cardiomyopathy [41], while efficacy remains uncertain in virus-positive disease [42].

Although direct assessment of cardiomyocyte contractile and diastolic function was beyond the scope of the present study, our transcriptomic data provide mechanistic insights into how the observed macrophage dynamics may contribute to functional impairment. At day 14, GSEA revealed significant negative enrichment of pathways related to muscle contraction and translation in macrophages, consistent with a disruption of the homeostatic macrophage–cardiomyocyte crosstalk that normally supports electrical conduction and metabolic homeostasis [11,12]. The pronounced expansion of Mac-rMHCII and its associated pro-inflammatory signature, including elevated *Il1b* and *Stat2* expression, may directly impair cardiomyocyte contractility through the cytokine-mediated suppression of calcium handling and sarcomeric protein function, which are mechanisms well-described in inflammatory cardiomyopathy [43]. Collectively, these findings suggest that macrophage transcriptional reprogramming during EAM progression creates conditions permissive to both systolic and diastolic dysfunction, although functional validation through echocardiography and cardiomyocyte-level assays will be required to confirm these mechanistic hypotheses.

In conclusion, our study delineates the temporal and functional heterogeneity of cardiac macrophages during EAM progression and identifies key immune–stromal interactions driving pathological remodelling. By uncovering subset-specific roles and niche-dependent signalling pathways, we provide a framework for the development of precision immunomodulatory therapies in myocarditis and its progression towards late cardiac remodelling.

## Data Availability

The datasets presented in this article are not readily available because the data are part of an ongoing study. Requests to access the datasets should be directed to Monika Stefańska.

## Supplementary Materials

The following supporting information can be downloaded at https://www.mdpi.com/article/10.3390/cells15121110/s1, Supplementary Figure S1: Comparative expression of key antigen-presentation genes between MHCII-positive macrophages and monocyte subsets; Supplementary Table S1: Differentially expressed genes identified in each cell subset by single-nuclei RNA sequencing analysis; Supplementary Table S2: Global ligand-receptor interactions predicted using LIANA; Supplementary Table S3: Benchmarking of the pydoubletfinder Python re-implementation; Supplementary Table S4: Single-cell expression values of MHC class II-related genes (Cd74, H2-Eb1, Ciita) in Classical Monocytes versus Resident Macrophage MHCII subsets, underlying the violin plot comparison shown in Supplementary Figure S1; Supplementary Table S5: Recovered Nuclei Per Sample.
