# Supplementary figures and images for: Cardiac Macrophages Exhibit Dynamic Heterogeneity and Functional Specialization During Experimental Autoimmune Myocarditis
Source: Cells. 2026 Jun 19;15(12):1110. doi: 10.3390/cells15121110 (PMC13296802; doi:10.3390/cells15121110)

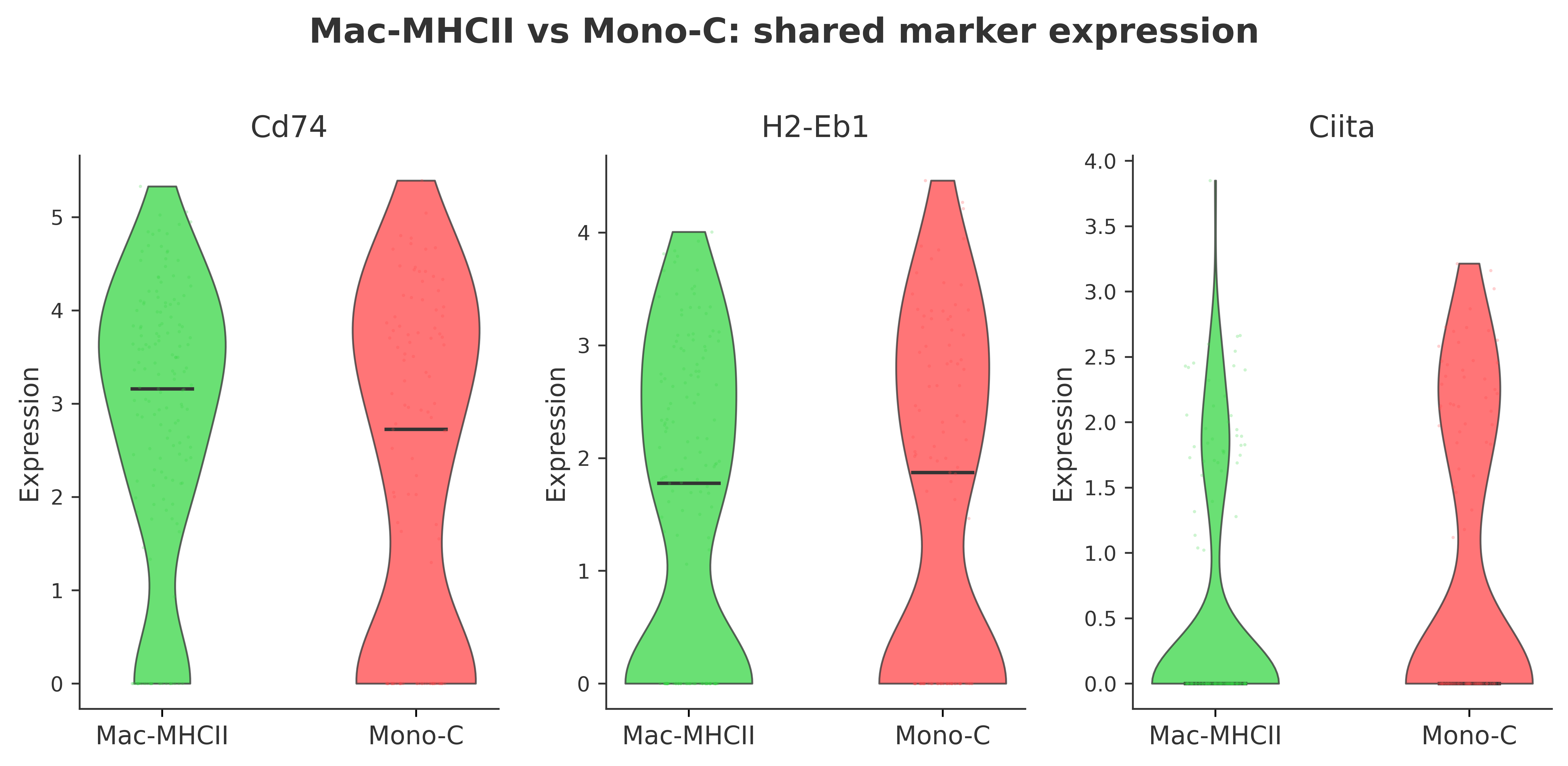

Supplement: Supplementary file 1 [file cells-15-01110-s001.zip › Suppl. Figure S1. violin_mac_mhcii_vs_mono_c.png]
